# Lipid Droplet Composition Varies Based on Medaka Fish Eggs Development as Revealed by NIR-, MIR-, and Raman Imaging

**DOI:** 10.3390/molecules25040817

**Published:** 2020-02-13

**Authors:** Ewelina Bik, Mika Ishigaki, Aneta Blat, Agnieszka Jasztal, Yukihiro Ozaki, Kamilla Malek, Malgorzata Baranska

**Affiliations:** 1 Faculty of Chemistry, Jagiellonian University, Gronostajowa 2, 30-387 Krakow, Poland; ewelina.bik@doctoral.uj.edu.pl (E.B.); aneta.blat@doctoral.uj.edu.pl (A.B.); 2 Jagiellonian Centre for Experimental Therapeutics (JCET), Jagiellonian University, Bobrzynskiego 14, 30-348 Krakow, Poland; agnieszka.jasztal@jcet.eu; 3 Raman Project Center for Medical and Biological Applications, Shimane University, 1060 Nishikawatsu, Matsue, Shimane 690-8504, Japan; ishigaki@life.shimane-u.ac.jp; 4 Faculty of Life and Environmental Sciences, Shimane University, 1060 Nishikawatsu, Matsue, Shimane 690-8504, Japan; 5 School of Science and Technology, Kwansei Gakuin University, 2-1 Gakuen, Sanda, Hyogo 669-1337, Japan; ozaki@kwansei.ac.jp

**Keywords:** near- and mid-infrared spectroscopic imaging, Raman spectroscopic imaging, lipid bodies, fertilized egg, lipids

## Abstract

In fertilized fish eggs, lipids are an energy reservoir for the embryo development and substrate for organogenesis. They occur in the cytoplasmic area and form lipid droplets (LDs), but also the yolk egg is composed of lipids and proteins. Insight on the LD formation and distribution and their interactions with other cellular organelles could provide information about the role based on the egg development. For non-destructive, macro-scale visualization of biochemical components of fish eggs, such as lipids proteins and water, near-infrared (NIR) imaging is the method of choice. Mid-infrared (MIR) and Raman spectroscopy imaging were used to provide details on chemical composition of LDs and other egg organelles. NIR imaging illustrated main compartments of the egg including membrane, LDs, yolk, relative protein, and lipid content in well-localized egg structures and their interactions with water molecules. In the yolk, a co-existence of lipids and proteins with carotenoids and carbohydrates was detected by Raman spectroscopy. Results showed a prominent decrease of unsaturated fatty acids, phospholipids, and triglycerides/cholesteryl esters content in the eggs due to the embryo development. An opposite trend of changes was observed by MIR spectroscopy for the glycogen, suggesting that consumption of lipids occurred with production of this carbohydrate. The comprehensive vibrational spectroscopic analysis based on NIR, MIR, and Raman imaging is a unique tool in studying in situ dynamic biological processes.

## 1. Introduction

Lipids are biomolecules with diverse biological roles such as an energy reservoir, signaling mediators and the main components of cellular membranes. Intracellularly, they are stored as fatty acids (FA), triacylglycerides (TG) and sterols in a form of lipid droplets (LDs) [1,2].

In fertilized fish eggs, lipids are energy reservoirs for the embryo development. During maturation, oil droplets in the cytoplasmic area of medaka *Oryzias latipes* eggs decrease in size and fuse to form one large oil droplet. Even just after hatching, the fish lava still possess a lipid globule in a body, which is absorbed in further development [3]. The yolk of *Oryzias latipes* eggs is composed of both lipids and proteins while oil droplets are mainly composed of unsaturated FA such as arachidonic acid, linoleic acid, docosahexaenoic acid and eicosapentaenoic acid [4,5].

Currently, insight on LD formation, distribution, and their interaction with other cellular organelles mainly rely on their visualization with the use of probes (hydrophobic dyes or immunochemical reactions) for fluorescence imaging [1,6]. However, staining with dyes is often unspecific since LDs are closely associated with surrounding membranous organelles and it is difficult to distinguish them from each other. To evaluate lipid droplet composition in various biological systems, gas chromatography is applied. Using such a method for lipid composition analysis is complicated because it requires sample preparation by extraction of lipids and use of standards of expected components present in the sample [7].

**Scheme 1:**
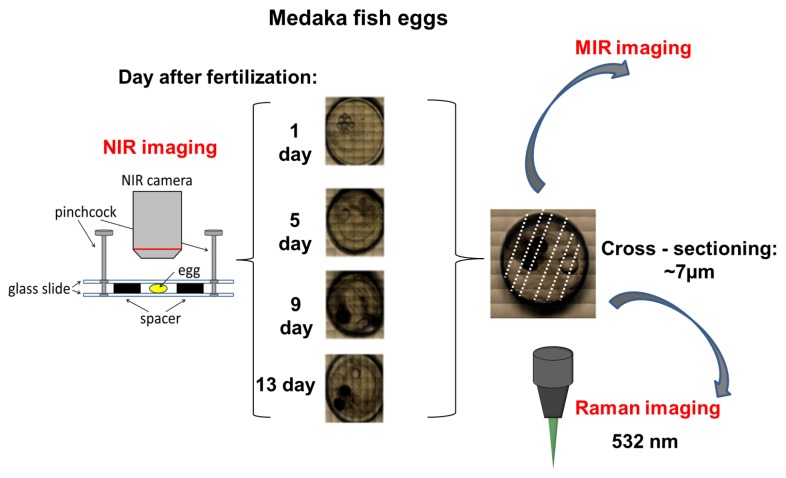
An overview of performed spectroscopic imaging of medaka *Oryzias latipes* eggs.

On the other hand, several approaches based on Raman and infrared spectroscopy are also used to image LDs. The most important advantage of both spectroscopes is that they provide clear and well-defined features of lipids in cells and tissues, which can be used to quantify lipids and to determine a degree of unsaturation and changes in fatty acid chains conformation, etc. [8,9,10,11,12]. Despite the fact that some reports described oil droplet size and their distribution in *Oryzias latipes* eggs, distinct changes in lipid components in developing eggs have not been identified yet [3,13,14,15]. Oil droplet visualization can be performed with use of these label-free spectroscopic techniques employing imaging mode. For non-destructive, visualization of biochemical components of fish eggs with a thickness of ca. 1 mm, near-infrared (NIR) imaging is the method of choice because of the possibility of examining thick samples such an eggs without special sample preparation. Based on NIR data, in previous study it was suggested that just before hatching, energy metabolism in *Oryzias latipes* eggs is changed [13]. Although NIR imaging enables measurement of a whole egg in a water environment and to determine biochemical components distribution, it is often difficult to interpret NIR spectra and to study subtle changes in chemical composition on contrary to mid-infrared (MIR) and Raman spectra that provide a fingerprint of molecules giving detailed information about the composition and molecular structure of a sample. However, illumination of a sample with MIR and laser (in Raman) light cannot penetrate sample as deeply as in NIR imaging, so to perform MIR and Raman imaging, thick samples need to be cross-sectioned [14]. Taking into account a diversity of molecular information gathered from Raman, NIR, and MIR spectra, we performed for the first time a deep analysis in macro- and micro-scale of distribution and composition of oil droplets in developing *Oryzias latipes* eggs (Scheme 1). Our work highlights the complementarity of these imaging techniques and shows the applicability of vibrational spectroscopy for investigation of lipid bodies. Strong points of NIR imaging can probe variations not only in proteins and lipids but also in water. The samples for NIR imaging do not require sample special preparation. This method can be used for thick samples, but thin samples such as cells cannot be measured with this method. Thick samples, such as fish eggs for Raman and MIR techniques, have to be sliced. However, Raman and MIR allow for collection of more detailed information about lipid from spectra, than NIR. Additionally, with the use of Raman, existence of carotenoids can be determined.

## 2. Results and Discussion

### 2.1. Near-Infrared (NIR) Imaging

**Figure 1:**
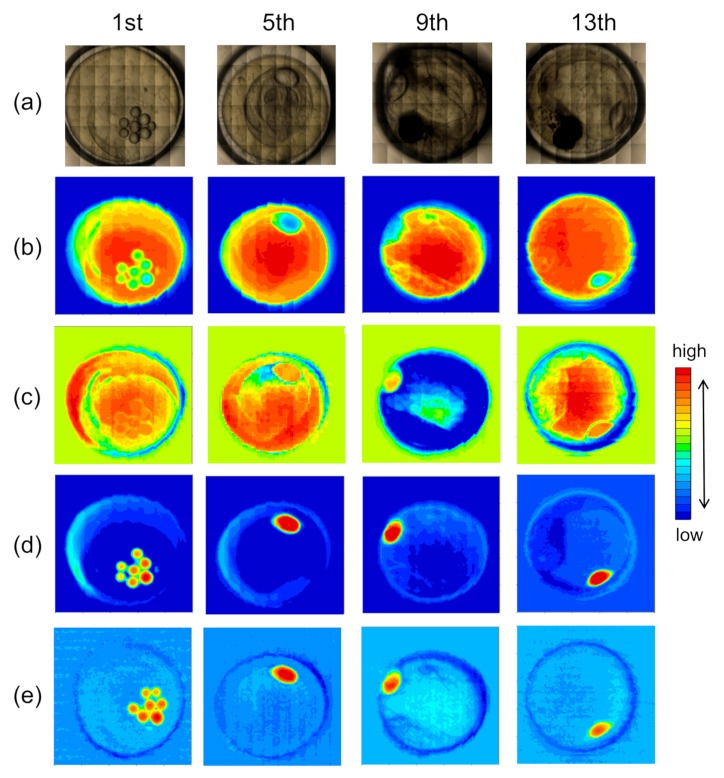
(**a**) Visible images of medaka fish eggs on the 1st, 5th, 9th, and 13th day after fertilization. NIR images constructed by (**b**) PC1, (**c**) PC2, (**d**) PC3, and (**e**) PC4 of principal component analysis (PCA) carried out for the dataset of second derivative NIR spectra including all developmental stages.

Figure 1a shows visible images of medaka fish egg on 1st, 5th, 9th, and 13th day after fertilization and NIR images made by PCs (principal components). NIR imaging was performed with spatial resolution of 25 μm. To extract the material variances in fish eggs depending on egg development, Principal Component Analysis (PCA) was carried out for the dataset of second derivative NIR spectra including 4 species of eggs at different stages (1st, 5th, 9th, and 13th days after fertilization). The values of each PCA score (PC1–PC4) were plotted in two dimensions and NIR images with PCA were constructed.

**Figure 2:**
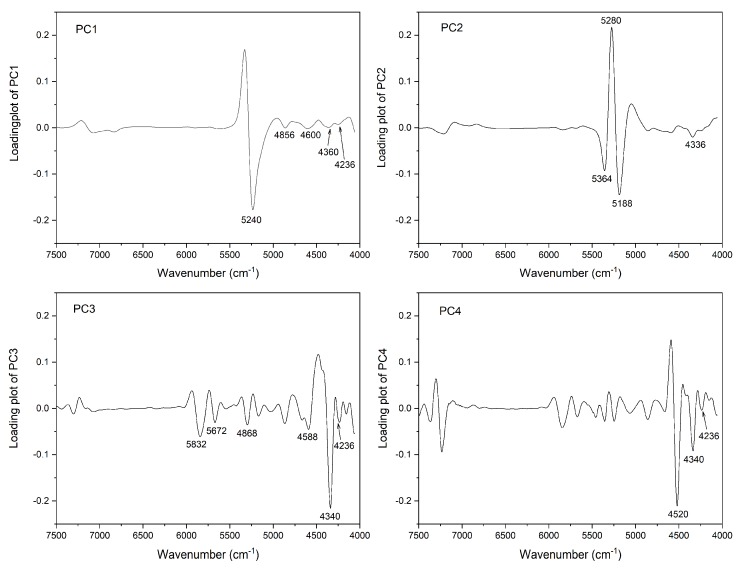
The loading plots of PC1, PC2, PC3, and PC4 as the result of PCA.

PC1 depicted the peak pattern of second derivative due to water (5250 cm^−1^), protein (4856 and 4600 cm^−1^), and lipid (4360 and 4236 cm^−1^) components in minus direction (Figure 2), making highlight the yolk part of the eggs (Figure 1b). PC2 showed three peaks in the wavenumber region of water absorbance (~5250 cm^−1^), which shifted the peak position due to water in lower wavenumber. A peak at 4336 cm^−1^ in PC2 assigned to C–H components, shown in Figure 2, indicated the variation of interactions between water and biomolecules. Yolk and oil droplet became clearer than embryonic body by PC2 (Figure 1c), which was consistent with the previous works by Ishigaki et al. [14,15]. The interesting image was constructed by PC3 and it seemed to show the component of unsaturated FA as representative peaks at 5832, 5672, 4588, 4340, and 4236 cm^−1^ (Figure 1d). The lipid components of unsaturated fatty acid seemed to seep out into the yolk over time as shown in Figure 1d, indicating that lipid components were used as nutrient for development. PC4 was likely to express the contribution of unsaturated FA, although egg membrane was dropped as a complementary relationship with PC3 (Figure 1e).

**Figure 3:**
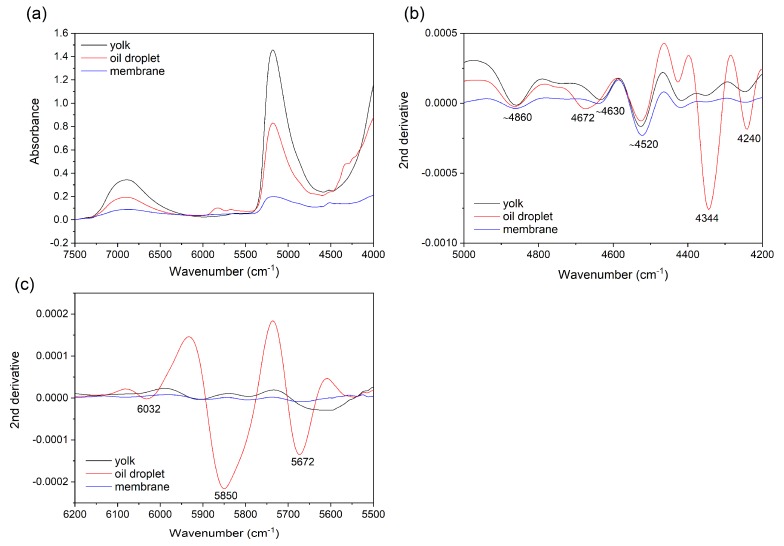
(**a**) NIR spectra and their second derivatives in the (**b**) 7500–4000 and (**c**) 5000–4200 cm^−1^ regions of yolk, oil droplet, and egg membrane of a medaka egg on the 1st day after fertilization.

Figure 3a depicts mean NIR absorbance spectra in the 7500–4000 cm^−1^ region measured for egg yolk, oil droplet, and egg membrane of a medaka fish egg on the 1st day after fertilization. Broad features due to water were observed at 6950 and 5200 cm^−1^, which were assigned to the combination of the antisymmetric and symmetric O–H stretching modes and that of the antisymmetric O–H stretching and O–H bending modes, respectively [16,17,18]. To capture the weak absorbance peaks due to biomolecules such as proteins and lipids, second derivative spectra were calculated. The second derivatives of the spectra shown in Figure 3a are enlarged in the 5000–4200 and 6200–5500 cm^−1^ regions in Figure 3b,c, respectively. Strong peaks due to lipids were observed at 4344 and 4240 cm^−1^ in the oil droplet (Figure 3b), which were attributed to the combination of the C–H stretching and bending modes of lipids [18,19,20]. Some weak peaks due to proteins (4860, 4630, and 4520 cm^−1^) were also detected in the second derivative NIR spectra of egg yolk and membrane (Figure 3b). They were assigned to combination modes of the N-H stretching and amide II (amide A + II), amide A, and amide III, to the amide mode of β-sheets in proteins, respectively [18,19,20,21,22,23,24]. Weak minima at 6032, 5850, and 5672 cm^−1^ appeared in the second derivative NIR spectra of unsaturated FA (Figure 3c) [19]; the first overtone of C–H stretching in unsaturated FA have significant peaks in that wavenumber region [25].

**Figure 4:**
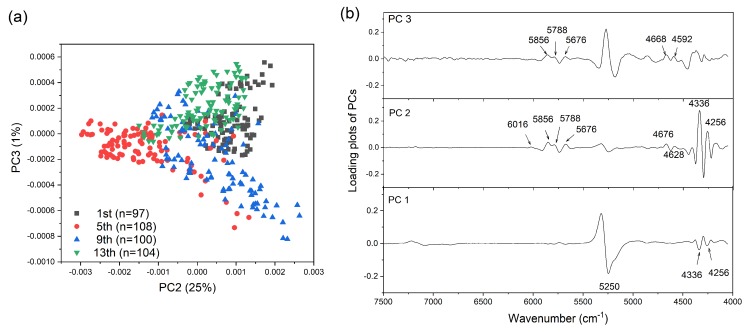
(**a**) A score plot of PC2 vs PC3 for the PCA carried out to the dataset of second derivative NIR spectra of oil droplets including all developmental stages (n: several spectra). (**b**) Loading plots of PC1, PC2, and PC3 of the PCA.

To extract the day-dependent variations in the spectra of oil components in the oil droplet and egg membrane, PCA was carried out for the dataset of second derivative NIR spectra including all developmental stages (1st, 5th, 9th, and 13th day after fertilization), as shown in Figure 4.

Figure 4a depicts a score plot of PCs 2 and 3. No systematic differences depending on the developmental stage were detected for the PC1 component. The score pattern showing roughly classification into four groups (Figure 4a) indicated that oil components varied with the development stage. Figure 4b expresses loading plots of PC1, PC2, and PC3. In the loading plot of PC1, average components due to oil (4336 and 4256 cm^−1^) and water (~5250 cm^−1^) within oil droplet appeared. In turn, the PC2 and PC3 loading plots showed some characteristic peaks arising from oil components, in particular, unsaturated FA (6200–5500 and 4700–4550 cm^−1^) with the combination modes of the C–H stretching and bending vibrations (4336 and 4256 cm^−1^) were clearly observed.

**Figure 5:**
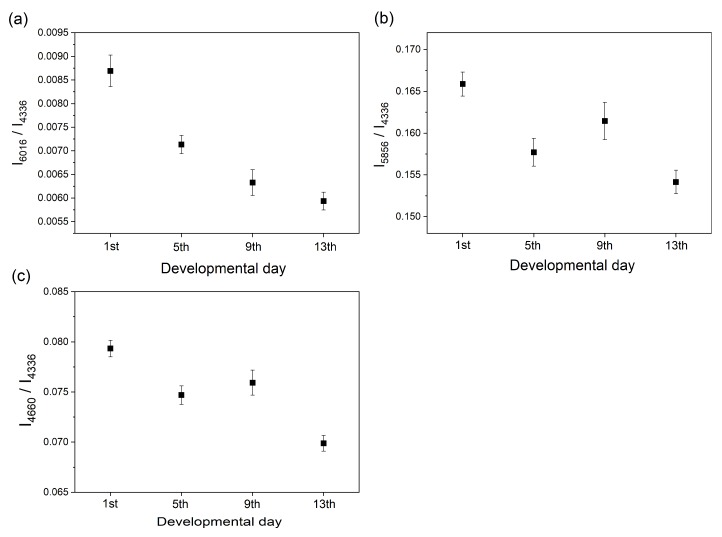
Variations in intensity ratios of two bands in the second derivative NIR spectra of (**a**) I_6016_/I_4036_, (**b**) I_5856_/I_4036_ and (**c**) I_4660_/I_4036_.

Generally, fish eggs have high contents of long-chain polyunsaturated FA such as docosahexaenoic and eicosapentaenoic acids [26]. In the NIR absorbance spectra of FA, the peak at ca. 5785 cm^−1^ assigned to the first overtone of C–H stretching modes shifts to a higher wavenumber in the spectra of unsaturated FA than in those of saturated ones [19,27,28], and as the degree of unsaturation increases the peak moves into a higher wavenumber. Grabska et al. calculated saturated and unsaturated long-chain fatty acid such as arachidonic acid, palmitic acid, stearic acid, linoleic acid, linolenic acid, and oleic acid by quantum chemical calculation, and they succeeded to reproduce spectral feature containing combination modes and to band assignment [28]. In fact, palmitic and stearic acids have the peak at 5785 cm^−1^, and it shifts to 5793, 5836, and 5842 cm^−1^ in the spectra of oleic, linoleic, and linolenic acid, respectively [28]. Furthermore, a small absorbance peak appears at around 6014 cm^−1^, which is the first overtone of the C–H stretches of the terminal methylene groups of vinyl and vinylidene structures [18]. The peak shift from 4672 cm^−1^ in saturated FA to 4660 cm^−1^ due to cis-unsaturation can also be a marker for investigating the degree of unsaturation [18,19]. In the loading plot of PC2, some peaks due to FA appeared at 6016, 5856–5676, and 4680–4628 cm^−1^, see Figure 4b. PCA results indicated the possibility of oil component variation with the change in the unsaturation degree depending on the developmental stages. To quantitatively evaluate the developmental day-dependent oil component variation, we investigated the proportions of the second derivative intensities of characteristic peaks of unsaturated FA at 6016, 5856, and 4660 cm^−1^ normalized by the one at 4336 cm^−1^. Figure 5a–c shows the decreasing patterns of ratios defined as (a) I_6016_/I_4336_, (b) I_5856_/I_4336_, and (c) I_4680_/I_4336_, i.e., the results indicated the decrement of the unsaturation degree of oil components in oil droplets with the egg development.

**Figure 6:**
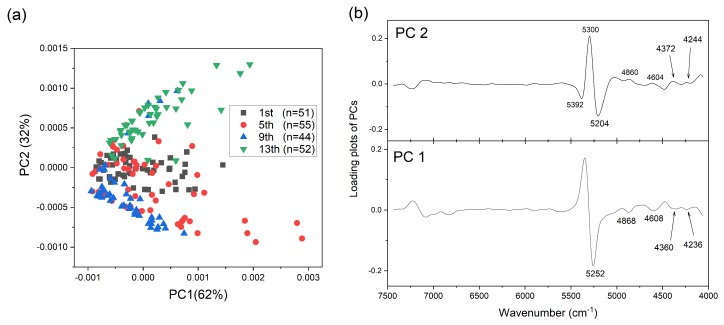
(**a**) Score plots of PC1 vs PC2 of PCA carried out to the dataset of egg membrane including all developmental stages. (**b**) Loading plots of PC1 and PC2 of PCA.

Similarly, a component variance depending on the development day within egg membrane was investigated. About 50 s derivative spectra obtained from the egg membrane for each stage were collected from different three eggs. PCA was carried out for the dataset of second derivative spectra including all four stages. Figure 6a depicts a score plot of PC1 vs PC2. In the loading plot of PC1 (Figure 6b), an average of second derivative spectral features for the egg membrane became clear, showing peaks due to water (5252 cm^−1^), proteins (4868 and 4608 cm^−1^), and lipids (4360 and 4236 cm^−1^).

Considering the shape of the egg membrane, the thickness at the measurement point was not uniform. Therefore, absorbance varied depending on the measurement location. The variance of PC1 was expected to reflect the thickness of the egg membrane. Moreover, as for PC2, the dataset of 9th and 13th were well classified as shown in Figure 6a. PC2 was the component to generate the gap from the second derivative spectra of egg membrane expressed by PC1. The PC2 score of 13th day biased in plus and the minus peaks in PC2 loading worked as a suppressive to the PC1. Therefore, the peak due to water at around 5250 cm^−1^ shifted to a lower wavenumber on the 13th day. The peaks of proteins (4860 and 4604 cm^−1^) and lipids (4372 and 4244 cm^−1^) appeared in a positive direction (Figure 6b). Therefore, the variation of the relative contribution due to proteins and lipids within the egg membrane may give the influence on water structure. Water interacts with the surrounding biomolecules such as proteins and lipids, and their compositional, concentration, and structural changes varies the interaction properties with water. In fact, the shape of the 5250 cm^−1^ water band in the second derivative spectrum was significantly different between the yolk and the oil droplets, indicating that the relative contribution of weakly and strongly hydrogen bonding water was different depending on contribution of surrounding water [13,28]. Furthermore, the data groups of the 1st, 5th, 9th days moved in minus direction PC2 overlapping each other. However, about the 13th day data, it was deposit on the opposite site of the 9th day in PC2. It is very interesting that the egg membrane comes to have completely different feature as nearing to hatch. In the previous work, published by Ishigaki et al., the water band in the second derivative NIR spectra obtained from the egg yolk drastically changed with the ones due to proteins and lipids on the day just before hatching. The big change enabled prediction of the hatching possibility with a very high accuracy, more than 99% [13]. In the present study, it was also made clear that the interaction between water and biomolecules was drastically changed near hatching.

### 2.2. Raman and Mid-Infrared (MIR ) Imaging

Cross-sections of a medaka fish egg from the 1st day after fertilization were examined with Raman and MIR imaging to study the distribution of different biochemical components. The egg cross-section with a diameter of ca. 1 mm was scanned with a step size of 10 μm to collected Raman image and a spatial resolution of 7.6 µm at 2500 cm^−1^ in MIR image. Therefore, we expected that the distribution of egg components in both chemical images was similar and better resolved than in NIR imaging (25 µm). Data collection time was ca. 1.5 h and 20 min for Raman and MIR imaging, respectively.

**Figure 7:**
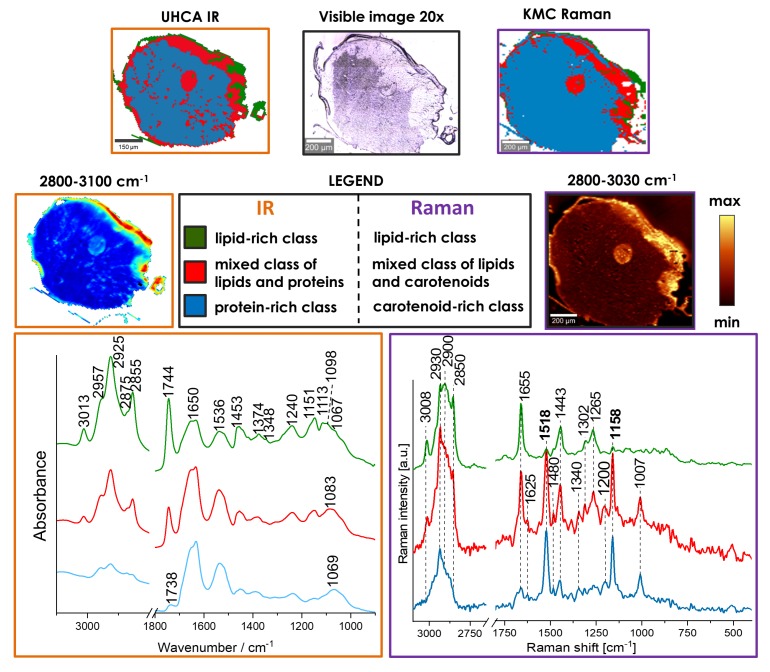
MIR (**left**) and Raman (**right**) images with their cluster analysis of a cross-section of a medaka fish egg collected on day 1 after fertilization; MIR and Raman spectra are mean spectra calculated in cluster analysis (the colors of spectra correspond to the colors of classes).

Figure 7 presents an example of Raman and Fourier-transform infrared (FTIR) imaging results of the egg cross-section from the 1st day of medaka fish development, showing its heterogeneous biochemical composition. Raman and IR chemical images of organic matter distribution in the sample were generated by integration of spectral regions within 2830–3030 and 2800–3100 cm^−1^, respectively, which contained bands originating from C–H stretching vibrations (mainly lipids and proteins). These Raman and IR images showed similar distribution of biomolecules and indicated distribution of lipid-rich egg structures on the periphery of the egg and in its center. To segregate chemical information k-means cluster analysis (KMC) and unsupervised hierarchical cluster analysis (UHCA) were employed for Raman and MIR images, respectively. UHCA discriminated three major classes: a lipid-rich class (green trace), a class of lipids with a minor contribution of proteins (red) and a protein-rich class (blue). However, Raman is very sensitive for carotenoids that is why all spectra are dominated by its characteristic bands. The green class is with the highest contribution of unsaturated lipids, blue is dominated by carotenoids, while red class is mixture of lipids and carotenoids (Figure 7). 

A class of lipids, extracted from KMC analysis of Raman hyperspectral data (green trace in Figure 3), was separated from the others by a characteristic spectral profile. Spectral features, which characterize lipids in the fingerprint region, are bands at 1302 and 1443 cm^−1^ assigned to deformations of long hydrocarbon chains, i.e., the twisting mode of the methylene group and the scissoring mode of the CH_2_/CH_3_ groups, respectively. The presence of bands at 3008, 1655 and 1265 cm^−1^ in spectra indicated vibrations of unsaturated C=C bonds [6]. A similar set of bands was found in the red trace and was accompanied by strong and very specific Raman features of carotenoids characteristic for 9 conjugated C=C bond polyene chain, i.e., the in-phase C=C stretching (1518 cm^−1^), the C–C stretching (1158 cm^−1^), and the CH_3_ wagging modes (1007 cm^−1^) [29]. These bands were observed in an almost whole area of the imaged cross-section (blue class) and this was in concordance with the fact that the egg from the first day after fertilization contains the carotenoids-rich yolk filling the egg [4,30]. Therefore, apart from the lipid-rich class with a low contribution of carotenoids, also protein and lipids mixed with carotenoid class was extracted during analysis. The presence of proteins was recognized i.a. by a narrow high-wavenumber region (2800–3030 cm^−1^) and lack of 3008 cm^−1^ band assigned unsaturated lipid vibration (blue trace in Raman spectra in Figure 7).

MIR spectra also identify lipids. The lipid-rich class with the minor contribution of proteins separated in UHCA analysis was defined based on several features of the mean spectrum (green trace in MIR spectra in Figure 7); bands at 2925 and 2855 cm^−1^ assigned to the antisymmetric and symmetric stretching vibrations of the CH_2_ groups (biomolecules containing long acyl chains) and a band at 1744 cm^−1^ (the stretching mode of the C=O group) attributed to cholesteryl esters and triglycerides [31,32]. Moreover, a week band at 3013 cm^−1^ originating from the C=H stretching vibrations of the olefinic groups and a band at 1240 cm^−1^ of the antisymmetric stretching vibrations of PO_2_^−^ groups clearly indicated the presence of unsaturated lipids and phospholipids in the lipid fraction [33]. The protein-rich class (blue) with the minor contribution of lipids indicated the presence of high intensity maxima of amide I (1650 cm^−1^) and amide II (1536 cm^−1^) bands [34]. The amide I band was split into two signals (1650 and 1633 cm^−1^) indicating the contribution of α-helical and β-sheet conformations of proteins. An UHCA class of mixed lipids and proteins (red class) without significant dominance of one of these components as indicated by relative intensities of these bands. Moreover, MIR spectra exhibited the presence of sugars as well as proteins (the region of 1200–1000 cm^−1^) that were evenly distributed in the egg. Among them we assigned the 1151 cm^−1^ band to glycogen whose high content was found in the lipid-rich fraction [31].

To sum up, both cluster analysis applied to Raman and MIR hyperspectral data showed the similar distribution of main components (lipids and proteins) of the egg cross-section by featuring the same number of extracted classes. However, a detailed analysis of mean Raman and IR spectra provided complementary information about additional components such as carotenoids and glycogen.

**Figure 8:**
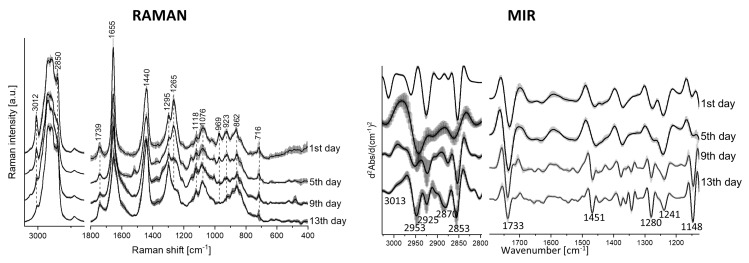
Averaged Raman (**left**) and second derivative MIR (**right**) spectra with standard deviation (±SD) for lipids of medaka fish eggs collected on the 1st, 5th, 9th, and 13th day after fertilization.

To study the dynamics of lipids composition in medaka fish eggs from 1st, 5th, 9th, 13th development day, only MIR and Raman spectra with clear lipids spectral feature were examined (Figure 8). The presence of bands originating from various lipid functional groups indicated the occurrence of mixture of lipids in oil droplets, which changed in the subsequent days of medaka fish egg development.

**Figure 9:**
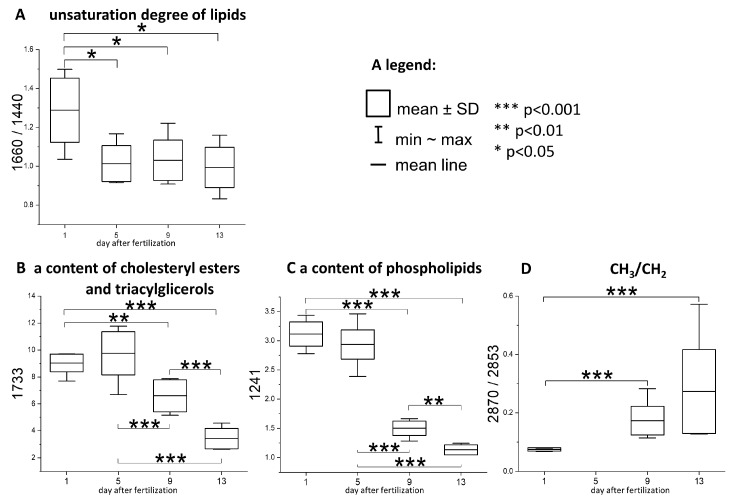
Selected spectral parameters showing alternations in composition of oil droplets in medaka fish eggs collected on the 1st, 5th, 9th and 13th after fertilization day: (**A**) Unsaturation degree of lipids—a ratio of intensities of the ν(C=C)/δ(CH_2_) bands (Raman: 1660/1440 cm^−1^); (**B**) A content of cholesteryl esters and triacylglycerols—intensity of the ν(C=O) band (MIR: 1733 cm^−1^); (**C**) A content of phospholipids—intensity of the ν_as_(PO_2_^−^) band (MIR: 1241 cm^−1^); (**D**) A ratio of intensities of the ν(CH_3_)/ν(CH_2_) bands (MIR: 2870/2853 cm^−1^).

The most pronounced spectral variations in the Raman spectra of were found for bands at 1265 cm^−1^ (δ(=CH)), 1655 cm^−1^ (ν(C=C)) and 3012 cm^−1^ (ν(=CH)) that decreased in intensity (Figure 8, left). Moreover, intensity of bands at 1295 cm^−1^ (τ(CH_2_)) and 1440 cm^−1^ (δ(CH_2_/CH_3_)) increased with the ongoing development of medaka fish egg. These changes were associated with alterations in unsaturation degree of acyl FA chains in lipids. To quantify it, we calculated the intensity ratio of the Raman bands at 1655 and 1440 cm^−1^ originating from the C=C and CH_2_ moieties, respectively (Figure 9A). A statistically significant decrease of the unsaturation degree of lipid appeared 5 days, after fertilization and the degree remained constant till the final stage of the egg development (*p* < 0.05, Tukey’s test, analysis of variance (ANOVA). An analysis of MIR spectra confirmed transformation of lipids due to the egg development (Figure 8, right). The most significant changes were found for the intensities of bands at 1733 and 1241 cm^−1^ corresponding to the content of triacylglycerols/cholesteryl esters and phospholipids. A level of these lipid components was expressed in a semi-quantitative manner by calculation of integral intensities of selected bands (Figure 9). The results showed a prominent decrease of phospholipids and triglycerides/cholesteryl esters content in the eggs due to the fish development on the 9th day (Figure 9B,C). While a gradual decrease of the esters level appeared from that day after fertilization, phospholipids significantly dropped down. A visual inspection of IR spectra displayed in Figure 8 also indicated an opposite trend of changes for the glycogen band (1148 cm^−1^), suggesting that consumption of lipids underwent with production of this carbohydrate. MIR bands at 2870 and 2853 cm^−1^ can express ratio between the terminal CH_3_ and chain CH_2_ groups of the acyl chains and were used to determine changes in the length of the fatty acid chains (Figure 9D; spectra from the 5th development day were affected by optimal cutting temperature medium (OCT) contribution which is mixture of mainly polyvinyl alcohol and polyethylene glycol so they were excluded). A slight increase of this ratio indicated the shortening of acyl FA chains near hatching since the intensity of the terminal methyl groups increased.

## 3. Materials and Methods

### 3.1. Sample Preparation

Japanese medaka (Oryzias latipes) was kept in fresh water at 25 °C. Eggs are spawned in the morning and they are immediately fertilized because spawning and insemination simultaneously occur. The fertilized medaka eggs were collected on the day they were laid. The day when eggs were laid was defined as the 1st day after fertilization. The eggs start their embryonic development soon and hatch at around two weeks after fertilization. The study was approved by the Ethics Committee of the Kwansei Gakuin University. The experiment was performed in accordance with the fundamental guidelines for proper conduct of animal experiment and related activities in academic research institutions under the jurisdiction of Ministry of Education, Culture, Sports, Science, and Technology in Japan.

NIR measurements were carried out for the eggs in vitro and in situ. For Raman and IR measurements on the contrary, eggs were fixed with the protocol published by European Molecular Biology Laboratory (EMBL Heidelberg, Germany) [35] as follows.

1. Separate embryos by rolling them gently in the petri dish or on a sheet of Whatman-paper.
2. Transfer separated embryos to a 25 mL glass vial and fix for 4 h at room temperature in 4% paraformaldehyde with 0.1% Tween20 in phosphate-buffered saline by vigorously rocking on a shaker.
3. Transfer embryos to a small petri dish.
4. Transfer embryos back to glass vial and wash 4 × 5 min in with 0.1% Tween20 in phosphate-buffered saline.
5. Wash 5 min at room temperature in 100% methanol.
6. Replace methanol and store embryos at least over night at −20 °C Comments: methanol treatment enhances probe penetration, a longer storage of embryos at −20 °C in methanol is advantageous. Set up fixed embryo stocks during periods of good egg laying.

For Raman and MIR imaging eggs were cross-sectioned because of a big thickness of the eggs, (1–1.5 mm). Cross-sectioning was performed on fixed eggs, using an electromechanical cryostat Leica CM 1950. Cryostat was equipped with a chamber, which allowed cutting frozen samples in minimal temperature of −35 °C. Eggs were embedded in OCT and frozen at −80 °C. Subsequently, cross-sections with a 7 μm thickness were taken from various heights of a sample and at least 10 of them were deposited on CaF_2_ windows.

### 3.2. NIR Imaging

NIR absorbance spectra were recorded in the 7800–4000 cm^−1^ region with an 8 cm^−1^ spectral resolution, a 25 μm spatial resolution, and 8 times accumulation. When NIR measurements of the eggs were carried out, they were fixed within two glass slides with pinchcock to regulate the optical path length as 0.36 mm. Special glass slides made of quartz were used for NIR measurements. Eggs were originally almost spherical in form, but they changed to irregular shapes a little by the procedure of NIR measurements. NIR absorbance spectra depending on the locations such as egg membrane and oil droplets were extracted from NIR imaging data by specifying the point within the egg. Savitzky-Golay method with 15 points was used for smoothing absorbance spectra and calculating second derivative spectra. For spectral analysis, the chemometrics software Unscrambler X 10.2 (Camo Analytics, Oslo, Norway), OriginPro 6.1 (OriginLab Corporation, MA, USA), and Graph-R free software were used.

### 3.3. MIR Imaging

FT-IR images were recorded using a Agilent 670-IR (United States) spectrometer combined with a 620-IR microscope and a liquid nitrogen cooled MCT FPA (Mercury Cadmium Telluride Focal Plane Array) detector comprising 16,384 pixels arranged in a 128 × 128 grid format. Transmission measurements were recorded with a 15× Cassegrain objective collecting 64 co-scans from a cross-section per pixel of the detector. All spectra were acquired in the range of 3800 to 900 cm^−1^ with a spectral resolution of 4 cm^−1^. The imaging area measured using this accessory and this FPA detector was ca. 700 μm × 700 μm (with a pixel size of 5.5 μm × 5.5 μm).

Prior to any analysis, the quality of each pixel-spectrum was evaluated using a sample thickness criterion according to intensity of amide I band (1620–1680 cm^‒1^). For all spectra, second derivative spectra were calculated using a Savitzky‒Golay algorithm with 13 smoothing points and were vector normalized in the spectral region of 1755–900 cm^−1^. Next, UHCA with a Ward’s algorithm was performed within the regions of 3020–2800 and 1755–900 cm^‒1^, and spectral distances were computed as D-Values. Data pre-processing and analysis were performed by using a Cytospec (ver. 2.00.01) software [36]. For further analysis, extracted mean spectra from images were used. 

Integral intensities of selected bands were computed in mean second derivative spectra drawing the straight line between the points of the two wavenumber limits defined and the area below this line was integrated. Box charts of bands integral intensities were constructed to show their mean value with standard deviation (SD) and min-max values. The groups were compared by using a statistical model ANOVA in the OriginPro 9.1 software (OriginLab, Northampton, Massachusetts, USA). Tukey’s test was employed to compute significance values (*p*).

### 3.4. Raman Imaging

Raman spectra were collected with the use of Confocal Raman Imaging system (WITec alpha 300) supplied with air objective with 20× magnification. The scattered light was directed to the spectrometer via 50 μm core diameter multimode fiber, which also acted as the pinhole for the confocal detection. The spectrometer was equipped with UHTS 300 spectrograph and Charge Coupled Device (CCD) detector (Andor, DU401A-BV-352) and 600 grooves/mm grating (BZL = 500 nm). For each measurement, a 532 nm laser was used with a power about 25 mW at the sample position. The Raman imaging was performed with a 10 µm step size and integration time of 0.5 s. Single Raman spectra were recorded with integration time of 0.5 s and co-adding of 10 accumulations.

Pre-processing of Raman data included cosmic spike removal and background subtraction (with the use of polynomial fitting 3rd order) k-Means cluster analysis (KMC) was performed for vector normalized spectra according to Manhattan (city block) method implemented in a WITec Project 5.0 Software. Integral intensities of selected bands were computed drawing the straight line between the points of the two wavenumber limits defined and the area above this line was integrated. Box charts of bands integral intensities were constructed to show their mean value with SD and min-max values. The groups were compared by using a statistical model ANOVA in the OriginPro 9.1 software (OriginLab, Northampton, Massachusetts, USA). Tukey’s test was employed to compute significance values (*p*).

## 4. Conclusions

The combination of three imaging techniques, NIR, MIR, and Raman spectroscopy, provided a comprehensive information about chemical composition of medaka eggs based on the analysis of fundamental modes and their overtones and combinations. The NIR imaging enabled non-destructive investigations in a water environment that illustrated the localization of the main compartments of the egg including membrane, oil droplets, and yolk. Despite the fact that the assignment of NIR bands to biomolecules was not straightforward and images required extensive chemometric analysis, interpretation of these spectra provided information about relative protein and lipid content in well-localized egg structures and their interactions with water molecules. The latter is a valuable result taking into consideration how difficult it is to assess in situ hydration state of macromolecules. MIR and Raman microscopic imaging gave chemical information at the micro-scale on the developing eggs from their cross-sections. The co-existence of lipids and proteins with carotenoids and carbohydrates as well as types of lipids were identified. During the medaka fish egg development, the size of yolk sphere rich in protein and lipids decreases, while oil droplets fuse into a single large globule located in the vegetal pole, whose content is finally absorbed in ca. 70% by the developing embryo. The content of unsaturated FA, phospholipids, and triacylglycerols decreased with various rates. Lipids in developing embryos provide energy until first. They are used for signaling, cellular membranes, and myelin formation. The decrement of unsaturation degree appeared already when the large oil globule was formed and then phospholipids and triacylglycerols were consumed by the developing embryo with a shortening of fatty acid chains in the advanced phase. Our results indicated that the comprehensive vibrational spectroscopic analysis, at macro- and micro-scale, can be a valuable tool for the observation of dynamic biological processes.
